# Depletion of Ars2 inhibits cell proliferation and leukemogenesis in acute myeloid leukemia by modulating the miR-6734-3p/p27 axis

**DOI:** 10.1038/s41375-018-0301-z

**Published:** 2018-12-05

**Authors:** Xiaoye Hu, Shuangnian Xu, Yibiao Chen, Ziyi Gao, Yunong Li, Jinjiao Hu, Xiuning Huang, Yanhao Zhang, Xiuxing Jiang, Lirong Li, Chong Yang, Jieping Chen, Ning Gao

**Affiliations:** 1 College of Pharmacy, Army Medical University, Chongqing, China; 2 Department of Hematology, Southwest Hospital, Army Medical University, Chongqing, China; 3 Greater Philadelphia Pharmacy, Philadelphia, PA USA; 4 Key Laboratory of Basic Pharmacology of Ministry of Education and Joint International Research Laboratory of Ethnomedicine of Ministry of Education, Zunyi Medical University, Zunyi, China

**Keywords:** Acute myeloid leukaemia, Oncogenes, Cancer genomics

## Abstract

Ars2 is a component of the nuclear cap-binding complex (CBC) that contributes to microRNA biogenesis and is required for cellular proliferation. Little is known regarding the functional role of Ars2 in cell proliferation and leukemogenesis of acute myeloid leukemia. Here, we show that the elevated expression of Ars2 was observed in acute myeloid leukemia (AML) cell lines and bone marrow samples from AML patients and was correlated with poorer overall survival. Overexpression of Ars2 promoted cell proliferation and colony formation in AML cells, whereas depletion of Ars2 inhibited cell proliferation and colony formation. Mechanistic studies reveal that depletion of Ars2 suppressed the interaction of Ars2 with CBC and led to alterations in miRNA processing. Furthermore, Ars2 depletion reduced the levels of miR-6734-3p, resulting in upregulation of p27 and culminating in cell cycle arrest at the G1 phase. In vivo studies indicate that depletion of Ars2 significantly reduced leukemic cell burden and prolonged the survival time of the leukemia-bearing mice. These findings indicate that Ars2 may not only play a crucial role in the regulation of cell proliferation and leukemogenesis, but could also be identified as a critical therapeutic target for treatment of AML.

## Introduction

Arsenic resistance protein 2 (Ars2) is an evolutionarily conserved gene that confers arsenite resistance in Chinese hamster ovary cells [1]. Ars2 is a nuclear protein that contains a number of domains, including an arginine-rich domain, a central RNA-binding domain, and a zinc-finger [2]. Biochemical studies indicate that Ars2 is a component of the nuclear cap-binding complex (CBC) that binds the 7-methylguanosine (7mG) cap structure of nuclear RNA polymerase II (RNAPII) transcripts [1, 3]. Ars2 interacts directly with the assembled CBP20/CBP80 cap complex to form a tertiary complex termed CBCA that stimulates mRNA 3′-end processing [4, 5]. Ars2 was reported to be highly conserved in mammals, zebrafish, and plants [6–9]. Ars2 deletion in metazoans is associated with developmental lethality in Drosophila, zebrafish, and mouse [6, 8]. Mutations of *SERRATE*, a plant gene with homology to Ars2, result in broad developmental defects [10, 11].

Recent evidence indicates that Ars2 is important for miRNA biogenesis and is required for cell proliferation [12]. Ars2 expression is linked to the proliferative state of the cells. Deletion of Ars2 led to a profound defect in cell proliferation and bone marrow failure in adult mice [6]. Ars2 may contribute to the stability and delivery of primary miRNA transcripts to the microprocessor complex in the nucleus. In *Arabidopsis*, mutations in *SERRATE*, a homolog of Ars2, decreased levels of mature miRNAs and increased levels of primary miRNA transcripts [13]. Ars2 directly interacts with Dicer-2 and facilitates the production of siRNAs in the cytoplasm [7]. In addition, Ars2 knockdown impairs miRNA-mediated silencing and reduces pri-miRNA transcript levels, suggesting a role for Ars2 in stabilizing pri-miRNAs [1, 14]. In mammalian systems, depletion of Ars2 from cell lines leads to reduced levels of miRNAs, including miR-21, miR-155 and let-7, implicated in transformation [1, 12, 15]. A biochemical study reveals that Ars2 is thought to bind directly to both the CBC component CBP80 and capped RNAs, leading to miRNA biogenesis. Direct interaction of Ars2 with Drosha is thought to link CBC-ARS2 to the miRNA processing pathway [7].

It has recently been reported that Ars2 is selectively expressed in proliferating cells, particularly in cancer cells, suggesting that Ars2 may be considered as a diagnostic and prognostic marker and a potential target for therapeutic intervention in various diseases, particularly in cancer. In addition, Ars2 was found to be overexpressed in human cholangiocarcinoma and hepatocellular carcinoma [16, 17]. Until now, the clinical significance of the Ars2 gene expression has not been explored in AML, nor have the mechanistic details of Ars2’s role in the regulation of miRNA biogenesis and leukemogenesis in AML been evaluated in depth.

In this study, we examined the expression of Ars2 in several AML cell lines and bone marrow mononuclear cells from AML patients. We found that elevated expression of Ars2 was observed in AML and was significantly associated with poor overall survival. We also provided the evidence that overexpression of Ars2 promoted cell proliferation and clonogenic growth of AML, whereas knockdown of Ars2 led to inhibition of cell proliferation and clonogenic growth of AML. By using miRNA mircoarray, we identified a novel miRNA, miR-6734-3p, that is a direct target miRNA of Ars2 and is critical for cell proliferation. We further identified that miR-6734-3p directly targets p27 which is essential for the regulation of cell cycle progression. These findings provide the evidence to support a potential role of Ars2 in the regulation of cell proliferation and leukemogenesis of AML and may identify Ars2 as a novel therapeutic target for treatment of AML.

## Materials and methods

### Patients sample and cell lines

Human leukemia cell lines U937, THP-1, HL-60, and K562 were purchased from American Type Culture Collection (ATCC, Manassas, VA). Cells were cultured in RPMI-1640 medium supplemented with 10% FBS. Bone marrow (BM) specimens from healthy donors and AML patients were collected, according to institutional guidelines and the declaration of Helsinki. Approval for these studies was obtained from the Southwest Hospital Institutional Review Board (Chongqing, China).

### MiRNA mimic, miRNA inhibitor, and shRNA

miRNA mimics and inhibitors for miR-6734-3p, miR-30e-3p, and miR-34c-5p were purchased from Ribobio (Guangzhou, China). Mimics and inhibitors were transfected using lipofectamine 3000 (Thermo Fisher Scientific, Waltham, MA, USA) for 24 h. Cells were stably transfected with a lentivirus vector expressing control shRNA (shCon) or shArs2 in the presence of 4 μg/ml polybrene and selected using 0.3 μg/ml puromycin for 72 h. shRNAs targeted to Ars2 were generated to the following sites, shArs2-1: 5′-GCGCAAACATATCTTCAACAA-3′ and shArs2-2: 5′-GCTGAGAATGACAGTTCTAAT-3′.

### RNA extraction and quantitative real-time PCR

Total RNA were extracted by RNAiso Plus reagent (TaKaRa, Dalian, China). Quantitative real-time PCR (qRT-PCR) analysis for determination of mRNA was performed by using one step SYBR PrimeScript^TM^ RT-PCR Kit (TaKaRa). The expression of β2-microglobulin (B2M) was used as the internal control. The sequence of primers for Ars2 were forward, GGTGACCTTCGACCGCAGTGTT; and reverse, TGGGTGATGCCGTTGATGTTGC. Primers for p27kip1 were forward, TAACCCGGGACTTGGAGAAG; and reverse, GCTTCTTGGGCGTCTGCTC. Primers for pri-miR-6734-3p were forward, TCTTGCAGATGGTTCGGGTG, and reverse, ACCCTTTCCCATAGTGGCCT. Primers for B2M were forward, CCTTGAGGCTATCCAGCGT; and reverse, CCTGCTCAGATACATCAAACATG. The levels of miRNA were determined by using Bulge-Loop^TM^ miRNA qRT-PCR system (Ribobio, Guangzhou, China) according to the manufacturer’s instructions. Primers of miRNAs (miR-6734-3p, miR-3751-3p, miR-101-5p, miR-449-5p, miR-548ah-3p, miR-548am-3p, miR-455-5p, miR-744-3p, miR-30e-3p, miR-34c-5p and U6) were designed RIBOBIO Corporation (Guangzhou, China). U6 small nuclear RNA was used as the internal control.

### Statistical analysis

Results repeated three times are expressed as mean ± SD. The comparisons were performed using Student’s *t*-test or one-way analysis of variance (ANOVA). *P < 0.05, **P < 0.01, and ***P < 0.001 were regarded as significant difference.

Additional Materials and Methods are presented in the Supplementary Data.

## Results

### High Ars2 expression is a prognostic indicator of poor survival in AML patients

**Fig. 1:**
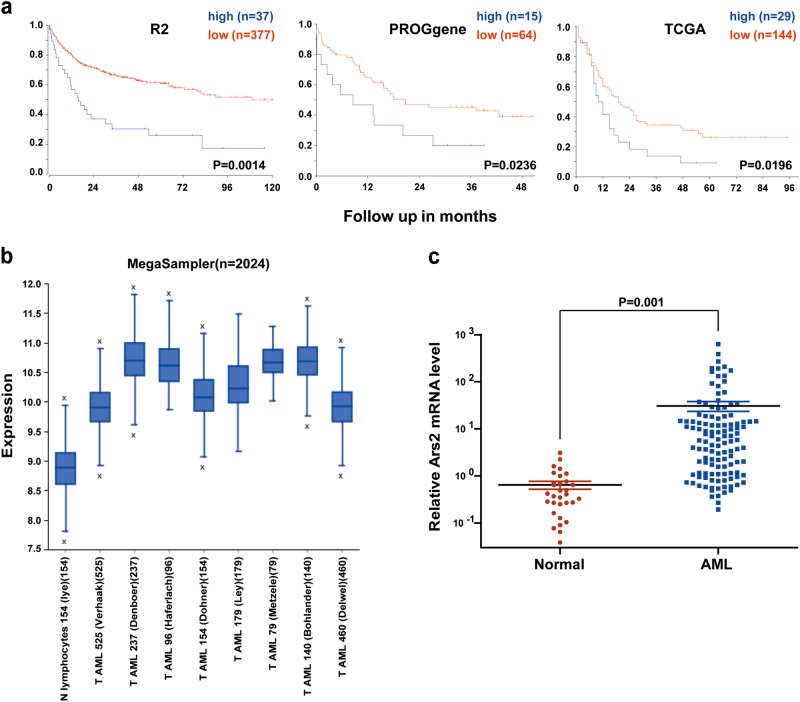
High Ars2 expression is a prognostic indicator of poor survival in AML patients. **a** Kaplan–Meier analysis of overall survival according to Ars2 expression for the R2, PROGgeneV2, and TCGA databases; *P* values were calculated with the log-rank test. **b** The expression of Ars2 was detected by R2 genomic analysis in 9 datasets, including 1 normal leukocytes/control dataset and 8 AML datasets as indicated. **c** The mRNA expression of Ars2 was detected by qRT-PCR analysis in mononuclear BM cells from 31 health donors and 120 AML patients. The significance was calculated with the non-paired Student *t* test (**P < 0.01)

To reveal the potential prognostic value of Ars2 mRNA expression for AML patients, we used genomic analysis and visualization platform databases (http://r2.amc.nl, http://watson.compbio.iupui.edu, and https://cancergenome.nih.gov). By using the R2, PROGgeneV2, and TCGA databases, we found that higher expression of Ars2 was significantly associated with decreased overall survival of AML patients (Fig. 1a). To verify whether Ars2 is upregulated in AML patients, R2 genomic analysis was performed to detect the mRNA expression of Ars2 in 9 datasets, including 1 normal leukocytes/control dataset and 8 AML datasets. Compared to normal leukocytes, Ars2 was significantly upregulated in AML patients (Fig. 1b). We also examined the expression of Ars2 at mRNA and protein levels in a variety of AML cell lines by using qRT-PCR and western blot analyses. In contrast to normal CD34^+^ bone marrow cells, elevated expressions of Ars2 at mRNA and protein levels were observed in all 4 AML cell lines (Supplementary Figures 1a and 1b). To further confirm the upregulation of Ars2 in AML patients, the bone marrow mononuclear cells from 31 healthy donors and 120 AML patients were collected and the mRNA expression of Ars2 was determined by qRT-PCR analysis. We found that the mRNA levels of Ars2 in AML patients were significantly higher than that in healthy individuals (Fig. 1c). These results highlight the clinical importance of Ars2 in determining the prognosis for AML patients.

### Overexpression of Ars2 promotes cell proliferation and colony formation in AML cells

**Fig. 2:**
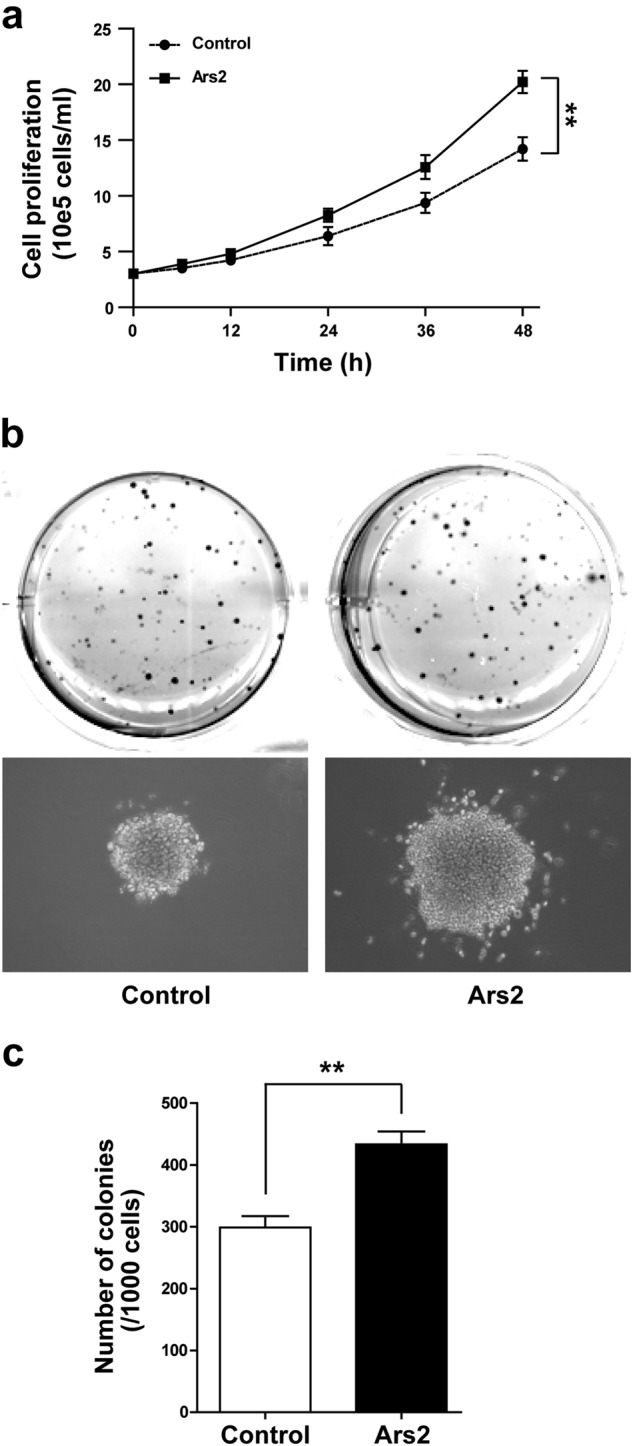
Overexpression of Ars2 promotes cell proliferation and colony formation in AML cells. U937 cells were transfected with vector control and Ars2. **a** U937 cells transfected with control or Ars2 were seed into a 12-well plate (3 × 10^5^ cells/ml), and cell proliferation was detected using cell counting with Beckman Coulter Z2 Particle Counter. Data represent the means ± SD from three independent experiments (*P < 0.05, **P < 0.01). **b**, **c** 1000 cells were mixed with 0.6% agar and 2-fold RPMI 1640 medium, and overlaid on 1.2% agar mixed with 2-fold RPMI 1640 medium in a 6-well plate. Colony formation was examined by staining colonies with 200 μl MTT per well. Colony number was counted using counter. The values represent the mean ± SD from three independent experiments. Values for Ars2-overexpressed cells are significantly higher than that for control cells by the Student’s *t* test; *P *<* 0.05 or **p < 0.01

To explore the functional role of Ars2 in the occurrence of AML, overexpression of Ars2 by lentivirus-mediated infection of U937 cells with either vector alone or Ars2 was employed. qRT-PCR and western blot analyses showed that the mRNA and protein levels of Ars2 in Ars2-overexpressing cells are more than 9-fold and 3-fold of those in vector control cells, respectively (Supplementary Figures 2a and 2b). Overexpression of Ars2 significantly promoted cell proliferation and colony formation compared with vector control cells (Fig. 2a, b, c). To determine whether these events were restricted to U937 cells, parallel studies were performed in multiple of AML cell lines including THP-1, HL60, and K562 cells. Significantly, the expressions of Ars2 at mRNA and protein levels in the cell lines overexpressed with Ars2 were higher than that in vector control cells (Supplementary Figures 3a and 3b). Overexpression of Ars2 significantly promoted cell proliferation and colony formation in comparison to vector control cells (Supplementary Figures 3c and 3d). These findings suggest that Ars2 could play an important role in the regulation of cell proliferation and leukemogenesis in AML.

### Depletion of Ars2 suppresses cell proliferation and colony formation in AML cells

**Fig. 3:**
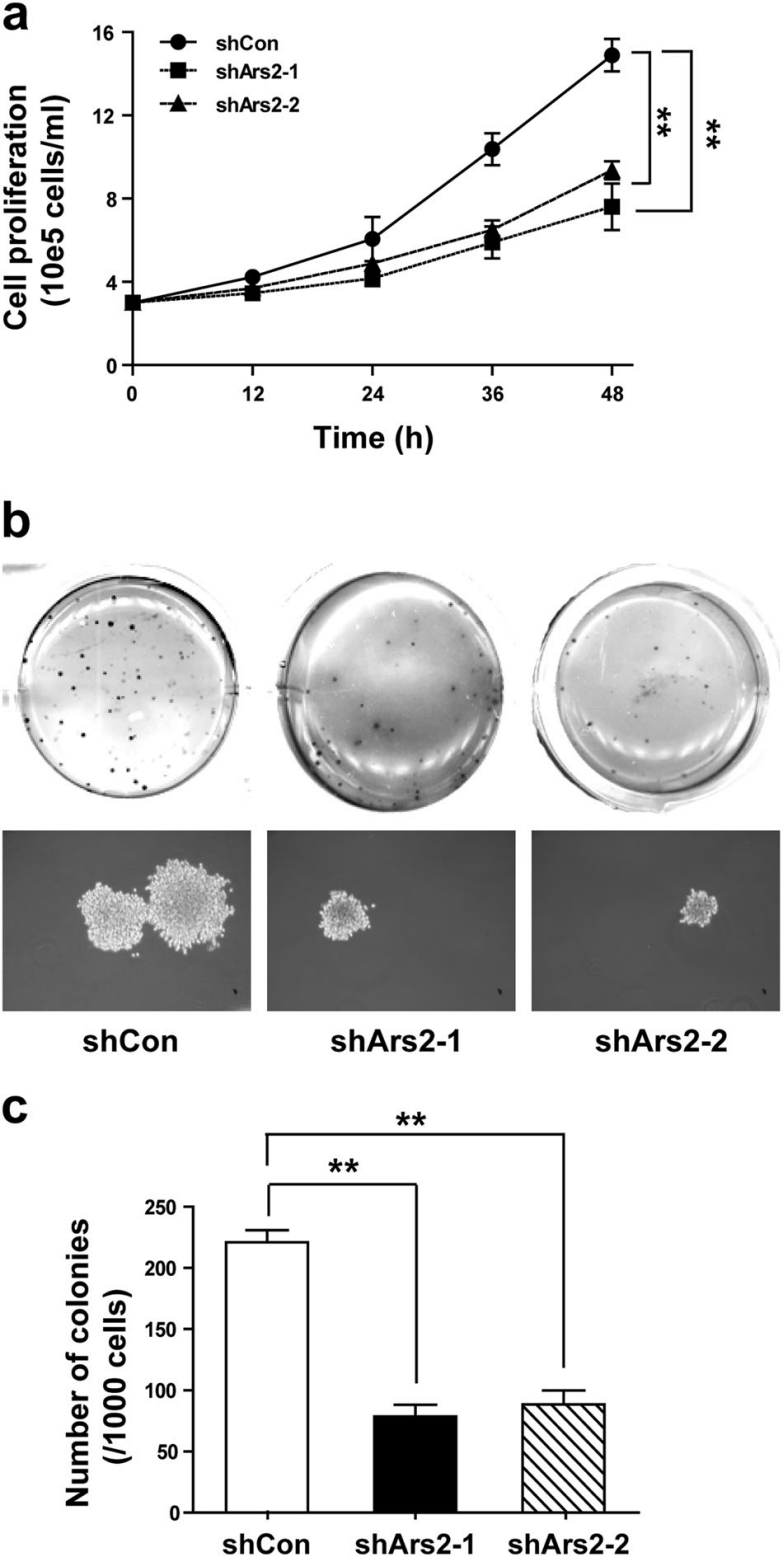
Depletion of Ars2 suppresses cell proliferation and colony formation in AML cells. U937 cells were transfected with vector control siRNA (shCon) and Ars2 siRNA (shArs2-1#, and shArs2-2#). **a** U937 cells transfected with shCon or shArs2 were seed into a 12-well plate (3 × 10^5^ cells/ml), and cell proliferation was detected using cell counting with Beckman Coulter Z2 Particle Counter. Data represent the means ± SD from three independent experiments (*P < 0.05, **P < 0.01). **b**, **c** 1000 cells were mixed with 0.6% agar and 2-fold RPMI 1640 medium, and overlaid on 1.2% agar mixed with 2-fold RPMI 1640 medium in a 6-well plate. Colony formation was examined by staining colonies with 200 μl MTT per well. Colony number was counted using counter. The values represent the mean ± SD from three independent experiments. Values for shArs2 cells are significantly lower than that for shCon cells by the Student’s *t* test; **P < 0.01

To further examine the role of Ars2 in cell proliferation and leukemogenesis in AML cells, two lentiviruses carrying shRNA (shArs2-1 and shArs2-2) were employed to stably knock down Ars2 expression in U937 cells. As determined by qRT-PCR and western blot analyses, U937 cells infected with Ars2 shRNA showed effective depletion of Ars2 at mRNA and protein levels compared to vector control (shCon) cells (Supplementary Figures 4a and 4b). Knockdown of Ars2 with shRNA noticeably suppressed cell proliferation and colony formation in comparison to shCon cells (Fig. 3a, b, c). We also examined the effects of depleting Ars2 with shRNA on cell proliferation and colony formation in other AML cell lines. shArs2-1 and shArs2-2 caused a near complete loss of detectable Ars2 mRNA and protein (Supplementary Figures 5a and 5b). Knockdown of Ars2 with shRNA significantly inhibited cell proliferation and colony formation in THP-1, HL60, and K562 cells compared to shCon cells (Supplementary Figures 5c and 5d). These findings further confirm the important role of Ars2 in regulating cell proliferation and leukemogenesis in AML.

### Depletion of Ars2 causes cell cycle arrest at G1 phase in U937 cells

**Fig. 4:**
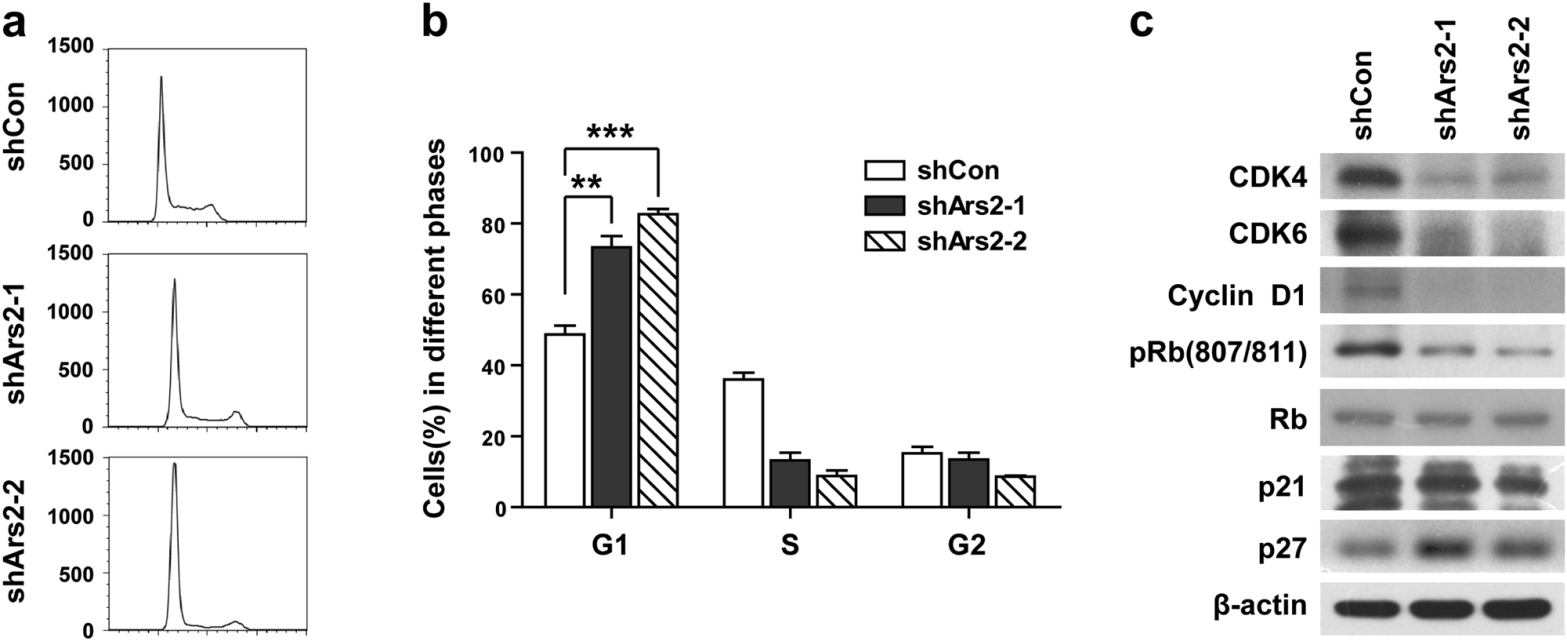
Depletion of Ars2 causes cell cycle arrest at G1 phase in U937 cells. U937 cells were transfected with shCon or shArs2. **a**, **b** Cell cycle analysis using propidium iodide (PI) and flow cytometry shows that Ars2 depletion increased the proportion of cells in G1 phase (**P < 0.01 compared to shCon cells). **c** G1 phase cell cycle related proteins as indicated were detected by western blot analysis

To explore the mechanism by which knockdown of Ars2 inhibits cell proliferation, we examined cell cycle distribution and apoptosis following Ars2 knockdown with shRNA by using flow cytometry. As shown in Fig. 4a, b, knockdown of Ars2 increased the accumulation of cells in the G1 phase in U937 cells, but did not increase apoptosis (data not shown). Consistent with these findings, knockdown of Ars2 decreased the expression of G1 cell cycle regulatory molecules including cyclin D, CDK4/6, and phospho-Rb (807/811) (Fig. 4c). To gain further insight into the mechanism of Ars2 knockdown-mediated cell cycle arrest at G1 phase, the expressions of p21 and p27 were determined by using western blot. Knockdown of Ars2 increased the expression of p27, but did not alter the expression of p21 (Fig. 4c). Collectively, these results indicate that knockdown of Ars2 inhibits cell proliferation through up-regulation of p27-mediated cell cycle arrest at G1 phase.

### Depletion of Ars2 suppresses biogenesis of miR-6734-3p

**Fig. 5:**
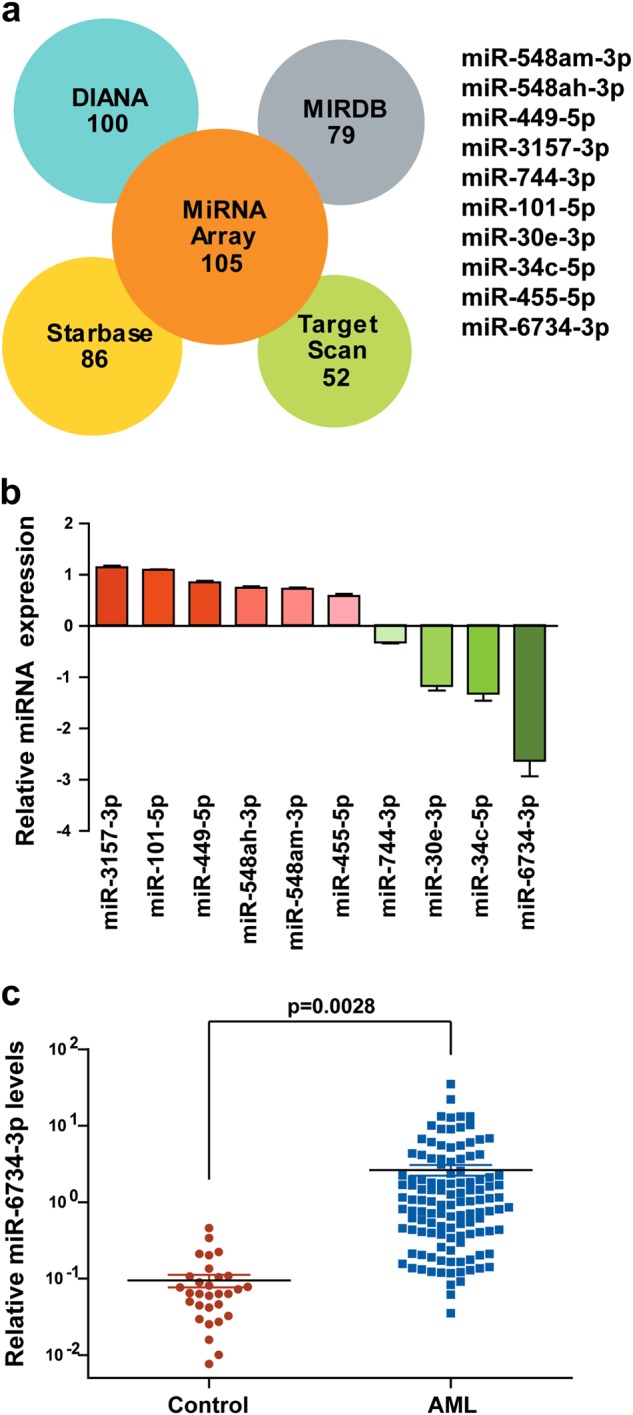
Depletion of Ars2 suppresses biogenesis of miR-6734-3p. U937 cells were transfected with shCon or shArs2. **a** The performing high-throughput screening of all genomic miRNAs was determined by using RiboArraymiDETECT MicroRNA Assay. Four microRNA target prediction databases (DIANA, MIRDB, Starbase, and Target Scan) were used to predict miRNAs which negatively regulate p27 expression. **b** The levels of 10 predicted miRNAs were validated by qRT-PCR analysis. **c** The expression of miR-6734-3p was detected by qRT-PCR analysis in mononuclear BM cells from 31 health donors and 120 AML patients. The significance was calculated with the non-paired Student *t* test (*P* = 0.0028)

Since Ars2 contributes to microRNA biogenesis under cell proliferation signaling [14], we subsequently analyzed the expression of miRNAs in U937 cells infected with shCon or shArs2 by performing high-throughput screening of all genomic miRNAs. We found that 105 miRNAs were significantly downregulated at least 2-fold (Supplementary Figure 6). It is likely that depletion of Ars2 induces upregulation of p27 through inhibition of miRNA, leading to cell cycle arrest at G1 phase. Therefore, we used four microRNA target prediction databases (DIANA [18, 19], MIRDB [20, 21], Starbase [21–23], and Target Scan [24, 25]) to find predicted miRNAs which negatively regulate p27 expression. Based on predicted miRNAs overlapped with miRNAs from microarray assays, we identified 10 potential down-regulated miRNAs (miR-3157-3p, miR-101-5p, miR-449-5p, miR-548ah-3p, miR-548am-3p, miR-455-5p, miR-744-3p, miR-30e-3p, miR-34c-5p, miR-6734-3p), which are related to upregulation of p27 (Fig. 5a). By using qRT-PCR analysis, only three miRNAs (miR-30e-3p, miR-34c-5p, miR-6734-3p) were down-regulated (>2-fold) after knockdown of Ars2 (Fig. 5b). To further confirm that Ars2 depletion-mediated downregulation of miR-30e-3p, miR-34c-5p, miR-6734-3p are involved in upregulation of p27, the mimics of these miRNAs were employed. Western blot analysis showed that treatment of cells with miR-6734-3p mimic decreased the expression of p27, whereas treatment of cells with miR-30e-3p and miR-34c-5p mimics did not affect the expression of p27 (Supplementary Figure 7), suggesting that only miR-6734-3p is involved in the regulation of p27.

To determine whether upregulation of Ars2 increases the expression of miR-6734-3p in AML patients, the bone marrow samples from 31 health donors and 120 AML patients were collected and the expression of miR-6734-3p was determined by qRT-PCR analysis. We found that the levels of miR-6734-3p in AML patients were significantly higher than that in health donors (Fig. 5c), suggesting that there is correlation between Ars2 and miR-6734-3p expression in AML. Together, these findings indicate that knockdown of Ars2 reduced the expression of miR-6734-3p, leading to upregulation of p27 and culminating in cell cycle arrest at the G1 phase.

### Ars2 interaction with CBC is required for biogenesis of miR-6734-3p

Increasing evidence reveals that Ars2 interaction with CBC is critical for miRNA biogenesis and cell proliferation [1, 5, 6]. To gain further insight into Ars2 function in cell proliferation of AML, immunoprecipitation of Ars2 followed by western blot analysis with Ars2, 20 kDa CBC subunit (CBP20), and CBP80 was employed. As shown in Supplementary Figure 8a, Ars2 was coimmunoprecipitated with CBP20 and CBP80 in shCon cells, and knockdown of Ars2 decreased the interaction of Ars2 with CBP20 or CBP80. Since the RNaseIII enzymes Drosha or Dicer interact with Ars2 to transform pri-miRNAs to mature miRNAs [3, 7, 26], we next determined the interaction of Ars2 with Drosha or Dicer by immunoprecipitation assay. Western blotting on these immunoprecipitates revealed that Ars2 was interacted with Drosha but not Dicer in shCon cells, and knockdown of Ars2 decreased the interaction of Ars2 with Drosha (Supplementary Figure 8a). To test whether miRNA maturation downstream of Drosha in the absence of Ars2, the levels of both pri-miR-6734-3p and mature miR-6734-3p were detected by qRT-PCR analysis. Depletion of Ars2 with siRNA led to increases in levels of pri-miR-6734-3p and decreases in levels of mature miR-6734-3p compared to shCon cells (Supplementary Figure 8b). These findings suggest that depletion of Ars2 may interrupt the cleavage of pri-miR-6734-3p, leading to the strong accumulation of pri-miRNA and reduction of mature miR-6734-3p.

### miR-6734-3p directly targets p27

**Fig. 6:**
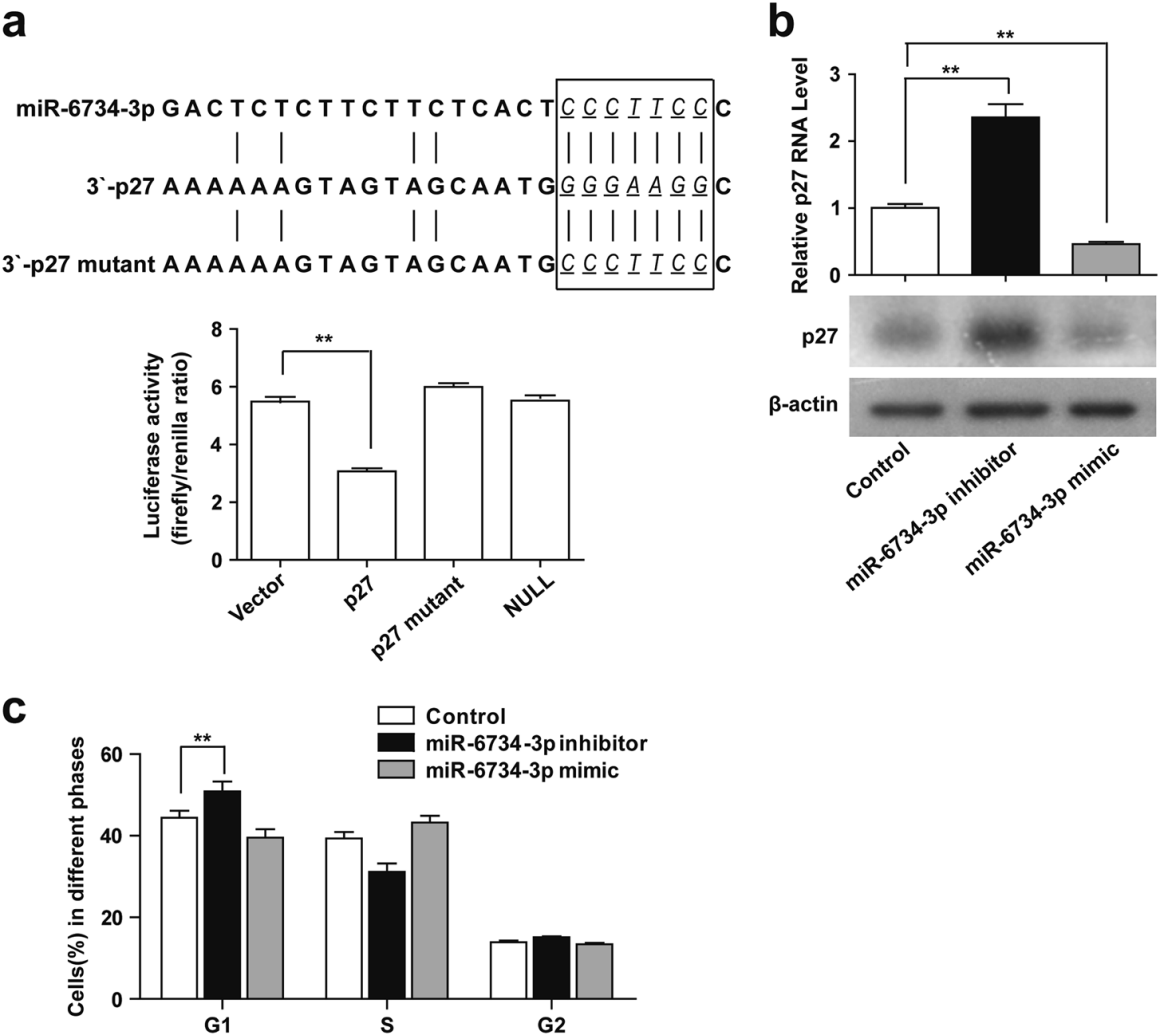
miR-6734-3p directly targets p27. **a** miR-6734-3p binding site on wild-type p27-3′UTR and mutant p27-3′UTR was predicted by RNA22. Dual-luciferase assay analysis for miR-6734-3p binding site; miR-6734-3p inhibited the activity of luciferase containing wild-type 3′UTR (**P < 0.001) but not that of luciferase containing mutant (mut) or null 3′UTR. **b** U937 cells were transfected without or with inhibitor or mimic of miR-6734-3p. The expression of p27 at mRNA and protein levels were detected by qRT-PCR and western blot analyses. Data were represented as mean ± SD for three independent experiments (**P < 0.01 compared with control). **c** Cell cycle distribution was determined by using propidium iodide (PI) and flow cytometry analysis, **P < 0.01 compared with control. Cell proliferation was detected using cell counting with Beckman Coulter Z2 Particle Counter, **P < 0.01 compared with control

To explore the possible mechanism by which p27 expression is negatively regulated by miR-6734-3p, we performed miRNA target site prediction using the RNA22 database (https://www.rna-seqblog.com/rna22-version-2-0-mirna-mre-predictions/) [27]. p27 was selected as a predicted miR-6734-3p target gene because of the well matched 3′-UTR binding sites by miR-6734-3p and its potential role in cell cycle progression (Fig. 6a). To confirm if miR-6734-3p binds to the 3′-UTR of p27, we cloned the 3′-UTR of p27 into a dual-luciferase vector. The dual-luciferase assay showed that miR-6734-3p inhibited luciferase activity with wt-p27-3′-UTR co-transfection compared with vector control, but did not influence luciferase activity with mut-p27-3′-UTR or null-p27-3′-UTR co-transfection (Fig. 6a). To further confirm whether p27 is a direct target of miR-6734-3p, miR-6734-3p mimics or inhibitor was employed. qRT-PCR and western blot analyses showed that inhibition of miR-6734-3p using inhibitor markedly increased the levels of p27, whereas overexpression of miR-6734-3p using mimics significantly decreased the levels of p27 compared with control (Fig. 6b). Flow cytometry analysis showed that inhibition of miR-6734-3p using inhibitor markedly increased percentage of cells at G1 phase, whereas overexpression of miR-6734-3p using mimics did not affect cell cycle progression (Fig. 6c). Cell counting showed that inhibition of miR-6734-3p using inhibitor markedly suppressed cell proliferation, whereas overexpression of miR-6734-3p using mimics induced cell proliferation (Supplementary Figure 9). Together, these findings suggest that miR-6734-3p directly targets p27, which regulates G1 cell cycle progression.

### Depletion of Ars2 inhibits leukemogenesis in vivo

**Fig. 7:**
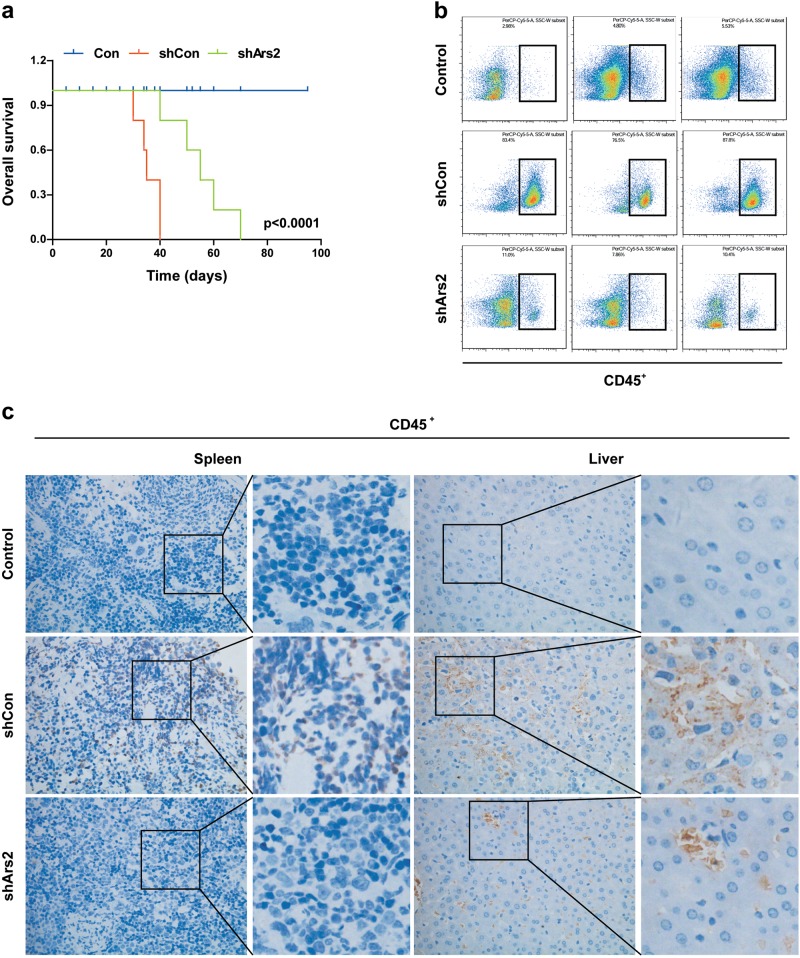
Depletion of Ars2 inhibits leukemogenesis in vivo. NOD/SCID mice were irradiated (8.5 Gy) and transplanted by tail-vein injection with normal saline (scramble), shCon-U937 cells, or shArs2-U937 cells. **a** Overall survival of mice transplanted with U937 cells expressing shCon and shArs2 or scramble (*n* = 10 mice per group). Statistical significance in survival was determined by log-rank test. *P < 0.01 compared with shCon group. **b** Tumor burden of BM was detected by flow cytometry (hCD45 staining). **c** Representative pictures of hCD45 staining from scramble, shCon and shArs2 mice

Since we found that knockdown of Ars2 suppresses cell proliferation and colony formation in U937 cells, we subsequently assessed whether knockdown of Ars2 could inhibit leukemogenesis in vivo by using leukemia xenograft mouse model. All NOD/SCID mice were irradiated at 8.5 Gy and transplanted by tail-vein injection with normal saline, shCon-U937 cells, or shArs2-U937 cells. As shown in Fig. 7a, mice transplanted with shCon-U937 cells had the shortest survival, whereas mice transplanted with shArs2-U937 cells had a prolonged overall survival. Mice transplanted with shCon-U937 cells also had severe splenomegaly, whereas the spleens of mice transplanted with shArs2-U937 cells appeared similar in size and weight compared with spleens of mice transplanted with saline control (Supplementary Figures 10a and 10b). Flow cytometry analysis showed that increased percentages (82.6%) of CD45^+^ cells in the BM of mice transplanted with shCon-U937 cells were observed, whereas low percentages (9.8%) of CD45^+^ cells in the BM of mice transplanted with shArs2-U937 cells were observed compared with saline control mice (4.4%) (Fig. 7b). Immunostaining of spleen and liver tissue slides with the proliferation marker CD45^+^ revealed that CD45^+^-positive cells were significantly higher in the spleens and livers of mice transplanted with shCon-U937 cells compared to that of saline control mice, whereas CD45^+^-positive cells were lower in the spleens and livers of mice transplanted with shArs2-U937 cells (Fig. 7c). Hematoxylin and eosin (HE) staining of spleen and liver sections of mice transplanted with shCon-U937 cells were infiltrated with leukemic cells, whereas the amounts of leukemic cells in these organs were lower in mice transplanted with shArs2-U937 cells (Supplementary Figure 11). Together, these findings suggest that knockdown of Ars2 could inhibit leukemogenesis in vivo.

## Discussion

Ars2 is a protein whose expression is strongly linked to the proliferation of cancer cells [28]. Recently, Ars2 was found to be overexpressed in human cholangiocarcinoma and hepatocellular carcinoma (HCC) and therefore considered as a diagnostic and prognostic marker, as well as a potential target for therapeutic intervention in cancer [15–17]. However, the clinical significance of Ars2 expression has not been explored in AML. In the present study, we found for the first time that overexpression of Ars2 was observed in AML patients and AML cell lines and is inversely correlated with overall survival of AML. First, by using R2 genomic analysis from 8 AML datasets, we found that Ars2 was highly expressed in AML patients. Second, by using qRT-PCR and Western blot analyses, we found that the expressions of Ars2 in AML patients and AML cell lines were higher than that in healthy donors and normal CD34^+^ bone marrow cells. Finally, by using the R2, PROGgeneV2, and TCGA databases, we found that higher expression of Ars2 was significantly associated with decreased overall survival of AML patients. The functional studies demonstrate that Ars2 plays a critical role in maintaining cell proliferation and clonogenic growth of AML. Overexpression of Ars2 promoted cell proliferation and colony formation, whereas knockdown of Ars2 suppressed cell proliferation and colony formation in AML. Furthermore, knockdown of Ars2 inhibited leukemogenesis in vivo. These findings suggest that Ars2 could emerge as a potential target for therapeutic intervention in AML. The clinical significance of Ars2 expression in AML warrants further investigation.

Ars2 is a component of the nuclear RNA cap binding complex (CBC) that is important for miRNA biogenesis and cell proliferation [1, 5]. Ars2 efficiently associates with both core components of the CBC, CBP80 and CBP20 [1, 29]. Ars2/CBC complex may recruit the Microprocessor complex (Drosha/Pasha) to nascent primary miRNA (pri-miRNA) transcripts, leading to pre-mRNA splicing [29–32]. Consistent with these reports, our results indicate that Ars2 can be immunoprecipitated with both core components of the CBC (CBP80 and CBP20) and Drosha in controls. Furthermore, depletion of Ars2 decreased the interaction of Ars2 with either CBC complex or Drosha. Such findings suggest that co-interaction of Ars2 with the CBC (CBP80 and CBP20) on one hand, and Drosha on the other, would thus naturally bring the Microprocessor complex into contact with the pri-miRNA and allow pre-miRNA processing.

MiRNAs are endogenously synthesized small non-coding RNAs that interact with proteins to regulate gene expression through cleavage or translational repression of target mRNAs [33, 34]. Recent evidence reveals that Ars2 contributes to miRNA biogenesis under cell proliferation signaling. After knockdown of Ars2 with siRNA, the ability of miRNAs to repress the expression of a luciferase reporter construct was significantly reduced. These effects are likely due to a defect in miRNA biogenesis as Ars2-depleted cells had decreased levels of a subset of miRNAs including miR-21, let-7, and miR-155 [1, 14, 15]. The present findings suggest that the functional role of Ars2 in processing of miRNAs in AML differs from that of previous reports. In this study, we did not observe decreased levels of miR-21, let-7, and miR-155 in AML after depletion of Ars2, thus raising the possibility that other miRNAs might be involved in Ars2-regulated cell proliferation in AML. More interestingly, we identified a novel miRNA, miR-6734-3p, whose processing might contribute to cell proliferation regulated by Ars2 based on the following evidence. First, using miRNA array and qRT-PCR analyses, the levels of miR-6734-3p were decreased (>2-fold) after knockdown of Ars2 in U937 cells. Second, the elevated expression of miR-6734-3p was observed and is correlated with overexpression of Ars2 in AML. Third, similar to the result indicated that depletion of Ars2 suppressed cell proliferation, and overexpression of Ars2 promoted cell proliferation, inhibition of miR-6734-3p using inhibitor markedly suppressed cell proliferation, whereas overexpression of miR-6734-3p using mimics promoted cell proliferation. Collectively, these findings suggest that miR-6734-3p represents a novel target miRNA of Ars2 which might contribute to cell proliferation in AML.

Ars2 is a regulator of RNA polymerase II transcript processing that likely contributes to the cell cycle progression. Indeed, microRNA, mRNA splicing, and replication-dependent histones are all important for cell cycle progression [1, 5, 7, 14]. It has been shown that knockdown of Ars2 led to a reduction of properly processed histone mRNA and protein, which impairs cell cycle progression through S phase [12, 35, 36]. Recent studies have shown that translational repression of target mRNAs by miRNAs is relieved upon exit from the cell cycle [37]. However, the functional role of miRNAs regulated by Ars2 in cell cycle progression is largely unknown. Based on our data, it appears that Ars2 is essential for miRNA processing and cell cycle progression. Differing from previous reports that knockdown of Ars2 caused cell cycle arrest at S phase, our findings indicate that knockdown of Ars2 led to accumulation of U937 cells in the G1 phase of the cell cycle, which was confirmed by downregulation of cyclin-Cdk complex: cyclin D and Cdk4/6 and dephosphorylation of Rb (Ser807/811) [38–41]. The upregulation of p27 is important for cell cycle arrest at G1 phase in shArs2-U937 cells. Notably, we identified p27 as a major direct target of miR-6734-3p based on the following evidence. First, RNA22 analysis predicts that the 3′-UTR of p27 are bound by miR-6734-3p. Second, the activities of the luciferase reporter cloned with wild-type 3′-UTRs of p27 was highly significantly decreased in U937 cells. Third, inhibition of miR-6734-3p using inhibitor markedly increased the luciferase activity of 3′-UTR of p27 and the expression of p27 at mRNA and protein levels, whereas overexpression of miR-6734-3p using mimics decreased the luciferase activity of the 3′-UTR of p27 and the expression of p27 at mRNA and protein levels. Fourth, inhibition of miR-6734-3p using inhibitor caused cell cycle arrest at G1 phase and inhibited cell proliferation. These findings suggest that miR-6734-3p directly targets the 3′-UTR of p27 that is responsible for cell cycle progression.

**Fig. 8:**
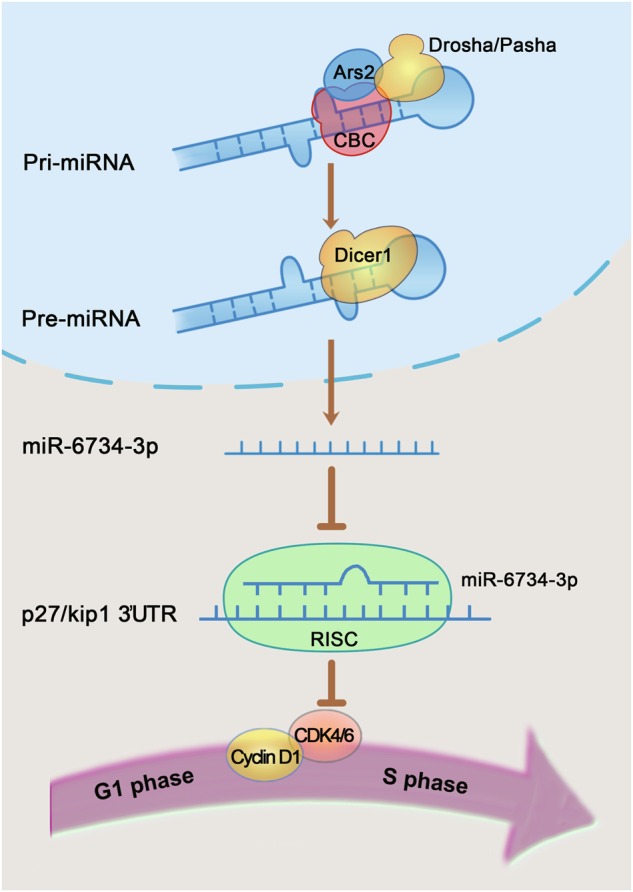
A model for Ars2-mediated processing of miR-6734-3p that targets p27^Kip1^-regulated G1 cell cycle progression

In summary, we revealed a novel mechanism supporting a critical role of Ars2 in cell proliferation and leukemogenesis in AML. Depletion of Ars2 reduced the level of miR-6734-3p by inhibiting the interaction of Ars2 with either CBC complex or Drosha, leading to p27 up-regulation-mediated G1 cell cycle arrest, and culminating in inhibition of cell proliferation and leukemogenesis in AML (Fig. 8). Our study could identify Ars2 as a potential therapeutic target for treatment of AML.

## Electronic supplementary material

Supplementary Information

## References

[1] Gruber JJ, Zatechka DS, Sabin LR, Yong J, Lum JJ, Kong M (2009). Ars2 links the nuclear cap-binding complex to RNA interference and cell proliferation. Cell.

[2] Grigg SP, Canales C, Hay A, Tsiantis M (2005). SERRATE coordinates shoot meristem function and leaf axial patterning in Arabidopsis. Nature.

[3] Gonatopoulos-Pournatzis T, Cowling VH (2014). Cap-binding complex (CBC). Biochem J.

[4] Andersen PR, Domanski M, Kristiansen MS, Storvall H, Ntini E, Verheggen C (2013). The human cap-binding complex is functionally connected to the nuclear RNA exosome. Nat Struct Mol Biol.

[5] Hallais M, Pontvianne F, Andersen PR, Clerici M, Lener D, Benbahouche Nel H (2013). CBC-ARS2 stimulates 3′-end maturation of multiple RNA families and favors cap-proximal processing. Nat Struct Mol Biol.

[6] Wilson MD, Wang D, Wagner R, Breyssens H, Gertsenstein M, Lobe C (2008). ARS2 is a conserved eukaryotic gene essential for early mammalian development. Mol Cell Biol.

[7] Nielsen AF, Gloggnitzer J, Martinez J (2009). Ars2 and the Cap-binding complex team up for silencing. Cell.

[8] Sabin LR, Zhou R, Gruber JJ, Lukinova N, Bambina S, Berman A (2009). Ars2 regulates both miRNA- and siRNA- dependent silencing and suppresses RNA virus infection in Drosophila. Cell.

[9] Voinnet O (2009). Fly antiviral RNA silencing and miRNA biogenesis claim ARS2. Cell Host Microbe.

[10] Dolata J, Taube M, Bajczyk M, Jarmolowski A, Szweykowska-Kulinska Z, Bielewicz D (2018). Regulation of plant microprocessor function in shaping microRNA landscape. Front Plant Sci.

[11] Mulligan MK, Dubose C, Yue J, Miles MF, Lu L, Hamre KM (2013). Expression, covariation, and genetic regulation of miRNA Biogenesis genes in brain supports their role in addiction, psychiatric disorders, and disease. Front Genet.

[12] O’Sullivan C, Christie J, Pienaar M, Gambling J, Nickerson PE, Alford SC (2015). Mutagenesis of ARS2 domains to assess possible roles in cell cycle progression and microRNA and replication-dependent histone mRNA biogenesis. Mol Cell Biol.

[13] Lepe-Soltero D, Armenta-Medina A, Xiang D, Datla R, Gillmor CS, Abreu-Goodger C (2017). Annotating and quantifying pri-miRNA transcripts using RNA-Seq data of wild type and serrate-1 globular stage embryos of Arabidopsis thaliana. Data Brief.

[14] Gruber JJ, Olejniczak SH, Yong J, La Rocca G, Dreyfuss G, Thompson CB (2012). Ars2 promotes proper replication-dependent histone mRNA 3’ end formation. Mol Cell.

[15] He Q, Cai L, Shuai L, Li D, Wang C, Liu Y (2013). Ars2 is overexpressed in human cholangiocarcinomas and its depletion increases PTEN and PDCD4 by decreasing microRNA-21. Mol Carcinog.

[16] He Q, Huang Y, Cai L, Zhang S, Zhang C (2014). Expression and prognostic value of Ars2 in hepatocellular carcinoma. Int J Clin Oncol.

[17] Cui L, Gao C, Zhang RD, Jiao Y, Li WJ, Zhao XX (2015). Low expressions of ARS2 and CASP8AP2 predict relapse and poor prognosis in pediatric acute lymphoblastic leukemia patients treated on China CCLG-ALL 2008 protocol. Leuk Res.

[18] Vlachos IS, Hatzigeorgiou AG (2017). Functional analysis of miRNAs using the DIANA tools online suite. Methods Mol Biol.

[19] Paraskevopoulou MD, Vlachos IS, Hatzigeorgiou AG (2016). DIANA-TarBase and DIANA suite tools: Studying experimentally supported microRNA targets. Curr Protoc Bioinform.

[20] Wang X (2008). miRDB: a microRNA target prediction and functional annotation database with a wiki interface. RNA.

[21] Wong N, Wang X (2015). miRDB: an online resource for microRNA target prediction and functional annotations. Nucleic Acids Res.

[22] Li JH, Liu S, Zhou H, Qu LH, Yang JH (2014). starBasev2.0: decoding miRNA-ceRNA, miRNA-ncRNA and protein-RNA interaction networks from large-scale CLIP-Seq data. Nucleic Acids Res.

[23] Yang JH, Li JH, Shao P, Zhou H, Chen YQ, Qu LH (2011). starBase: a database for exploring microRNA-mRNA interaction maps from Argonaute CLIP-Seq and Degradome-Seq data. Nucleic Acids Res.

[24] Agarwal V, Bell GW, Nam JW, Bartel DP. Predicting effective microRNA target sites in mammalian mRNAs. *eLife* 2015; 4:e05005. 10.7554/eLife.05005. 10.7554/eLife.05005 PMC4532895 26267216

[25] Garcia DM, Baek D, Shin C, Bell GW, Grimson A, Bartel DP (2011). Weak seed-pairing stability and high target-site abundance decrease the proficiency of lsy-6 and other microRNAs. Nat Struct Mol Biol.

[26] Macias S, Cordiner RA, Caceres JF (2013). Cellular functions of the microprocessor. Biochem Soc Trans.

[27] Loher P, Rigoutsos I (2012). Interactive exploration of RNA22 microRNA target predictions. Bioinformatics.

[28] Bartel DP (2004). MicroRNAs: genomics, biogenesis, mechanism, and function. Cell.

[29] Giacometti S, Benbahouche NEH, Domanski M, Robert MC, Meola N, Lubas M (2017). Mutually exclusive CBC-containing complexes contribute to RNA fate. Cell Rep.

[30] Perales R, Bentley D (2009). “Cotranscriptionality”: the transcription elongation complex as a nexus for nuclear transactions. Mol Cell.

[31] Lee M, Kim B, Kim VN (2014). Emerging roles of RNA modification: m(6)A and U-tail. Cell.

[32] Han J, Lee Y, Yeom KH, Nam JW, Heo I, Rhee JK (2006). Molecular basis for the recognition of primary microRNAs by the Drosha-DGCR8 complex. Cell.

[33] Carthew RW, Sontheimer EJ (2009). Origins and mechanisms of miRNAs and siRNAs. Cell.

[34] Ebert MS, Sharp PA (2012). Roles for microRNAs in conferring robustness to biological processes. Cell.

[35] Sabath I, Skrajna A, Yang XC, Dadlez M, Marzluff WF, Dominski Z (2013). 3’-End processing of histone pre-mRNAs in Drosophila: U7 snRNP is associated with FLASH and polyadenylation factors. RNA.

[36] Kiriyama M, Kobayashi Y, Saito M, Ishikawa F, Yonehara S (2009). Interaction of FLASH with arsenite resistance protein 2 is involved in cell cycle progression at S phase. Mol Cell Biol.

[37] Vasudevan S, Starostina NG, Kipreos ET (2007). The Caenorhabditis elegans cell-cycle regulator ZYG-11 defines a conserved family of CUL-2 complex components. EMBO Rep.

[38] Patel P, Tsiperson V, Gottesman SRS, Somma J, Blain SW (2018). Dual inhibition of CDK4 and CDK2 via targeting p27 tyrosine phosphorylation induces a potent and durable response in breast cancer cells. Mol Cancer Res.

[39] Blain SW (2018). Targeting p27 tyrosine phosphorylation as a modality to inhibit CDK4 and CDK2 and cause cell cycle arrest in breast cancer cells. Oncoscience.

[40] Sawai CM, Freund J, Oh P, Ndiaye-Lobry D, Bretz JC, Strikoudis A (2012). Therapeutic targeting of the cyclin D3: CDK4/6 complex in T cell leukemia. Cancer Cell.

[41] Ferguson KL, Callaghan SM, O’Hare MJ, Park DS, Slack RS (2000). The Rb-CDK4/6 signaling pathway is critical in neural precursor cell cycle regulation. J Biol Chem.

