# Supplementary material for: Depletion of Ars2 inhibits cell proliferation and leukemogenesis in acute myeloid leukemia by modulating the miR-6734-3p/p27 axis
Source: Leukemia. 2018 Dec 5;33(5):1090–101. doi: 10.1038/s41375-018-0301-z (PMC6756072; doi:10.1038/s41375-018-0301-z)
Supplement: Supplementary file 1 — Supplementary Information [file 41375_2018_301_MOESM1_ESM.docx]

**Supplementary Data**

**Supplementary Materials and Methods**

**Reagents**

Antibodies against Ars2, CDK4, CDK6, Cyclin D, CBP20, CBP80, Dicer, Drosha, and β-actin were purchased from Santa Cruz Biotechnology (Santa Cruz, CA); phospho-Rb (Ser807/811), Rb, p21, and p27 were from Cell Signaling Technology (Beverly, MA); IgG was from Beyotime Biotechnology (Shanghai, China).

**MicroRNA Array and database analysis**

Total RNA TRIzol solution of shCon-U937 and shArs2-U937 were prepared for RiboArray (Ribobio), Hybridization Protocol was Ribobio miRNA Hybridization Protocol. Data annotation is based on the miRbase database release 21.0. Matching score > 0.8 miRNAs were selected in DIANA TOOLS (http://diana.imis.athena- innovation.gr/DianaTools/index.php). The all predicted miRNAs which could match 3` UTR of p27/kip1 were collected in TargetScan (<http://www.targetscan.org/vert_72/>), miRDB (<http://www.mirdb.org/cgi-bin/search.cgi>), starbase (<http://starbase.sysu.edu.cn/>).

**Soft agar and colony formation**

Sustainment gel was mixed 0.6% agarose (low gelling temperature, Sigma-Aldrich (St. Louis, MO, USA)) in 1640 medium 1 ml/well in 12-well plates. Cultivate gel was mixed 0.3% agarose in 1640 medium with 10% FBS. 1×10^3^ cells were cultured in 1 ml cultivate gel above concretionary sustainment gel. After 21 days, the colonies were photographed and the number of colonies each well was counted.

**Luciferase assays**

U937 cells were transfected in 24-well plates using Lipofectamine 3000 (Invitrogen) according to the manufacturer’s protocol. The transfection mixtures contained 100 ng of firefly luciferase reporter plasmid, 400 ng of plasmids expressing wt-3′-UTR or mut-3′-UTR of p27/kip1. pRL-TK (Promega) was also transfected as a normalization control. Cells were collected 48 h after transfection, and luciferase activity was

measured using a dual-luciferase reporter assay system (Promega). All experiments were performed in triplicate and at least three times independently.

**Cell cycle analysis**

For cell cycle analysis, cells were harvested and washed twice with PBS. Then cells were stained with prepared nuclei staining buffer (0.1% Triton X-100 in PBS, 200 g/ml RNase, and 20 g/ml PI) for 2 h at 4°C. Cell cycle histograms were generated by FACS with flowjo (Version 7.6; TreeStar, Ashland, OR, USA). For each sample, at least 2×10^4^ events were recorded. Histograms generated by FACS were analyzed by ModFit Cell Cycle Analysis software (Verity, Topsham, ME) to determine the percentage of cells in each phase (G1, S, and G2/M).

**Western blot analysis**

Cells were lysed on ice for 30 min in RIPA buffer (Beyotime, Shanghai, China). The total cellular protein extracts were separated by SDS-PAGE and transferred to nitrocellulose membrane in 20 mM Tris–HCl (pH 8.0), containing 150 mM glycine and 20% (vol/vol) methanol. The membrane was blocked with 5% nonfat dried milk, and then incubated with primary antibodies overnight at 4℃. The bands were washed by TBST for three times, and incubated with horseradish peroxidase-conjugated antibodies (Kirkegaard and Perry Lboratories, Gaithersoburg, MD, USA). At last, these bands were visualized with Clarity Western ECL Substrate (Bio-Rad, Hercules, California, USA).

**Immunoprecipitation assay**

For immunoprecipitation, total protein lysates was obtained as described, equal quantities of proteins were incubated with primary antibodies at 4°C on a rocking plat-form. Immune complexes were collected with protein A/G agarose beads (Beyotime, Shanghai, China) followed by 5 times wash in PBS, samples were subjected to SDS-PAGE and Western blot.

**Murine xenograft model**

Animal studies were performed according to federal guidelines and approved by the Army Medical University Institutional Animal Care and Use Committee. NOD/SCID mice (5 weeks old) were obtained from Vital River Laboratories (Beijing, China). Mice were divided to three group including normal saline, shCon-U937 and shArs2-U937, 10 mice per group. Mice were exposed to 1.5 Gy of irradiation 70s. 24 h later, mice were transplanted intravenously with normal saline, shCon-U937 and shArs2-U937 cells (200 μl of 3×10^7^ cells/ml). Survival time of each group was recorded. The percentage of CD45^+^ cells from bone marrow of mice was determined by flow cytometry. Spleens and livers were fixed by formalin and embedded in paraffin. Hematoxylin and eosin (HE) staining and immunohistochemical analyses were employed for histopathological evaluation and determination of CD45^+^ expression.

**Supplementary Figures**

**
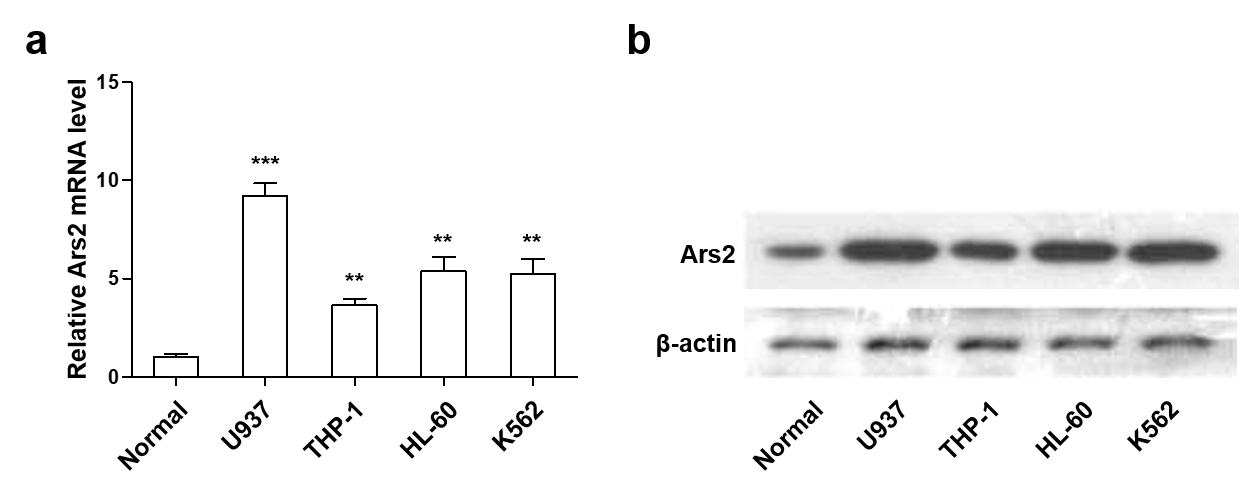
**

**Supplementary Figure 1.** High expression of Ars2 in AML cell lines. (a) The expression of Ars2 at mRNA levels in U937 cells was detected by qRT-PCR. Data from three independent experiments were normalized to B2M. **Values in AML cell lines are significant higher than that in normal CD34+ BM cells by the Student’s t test; *P* < 0.01. (b) The expression of Ars2 at protein was detected by western blot analysis.

**
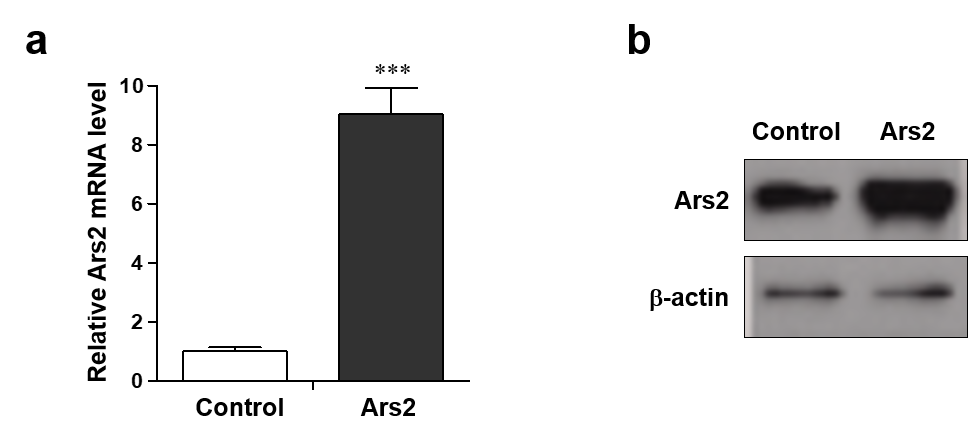
**

**Supplementary Figure 2.** Elevated expression of Ars2 in Ars2 overexpressing cells. U937 cells were transfected with either vector control or Ars2. (a) The expression of Ars2 at mRNA levels was determined by qRT-PCR analysis. Data from three independent experiments were normalized to B2M. ***Values for cells transfected with Ars2 are significantly higher than that in vector control cells (*P* < 0.001). (b) The expression of Ars2 at protein levels was detected by western blot analysis.

**
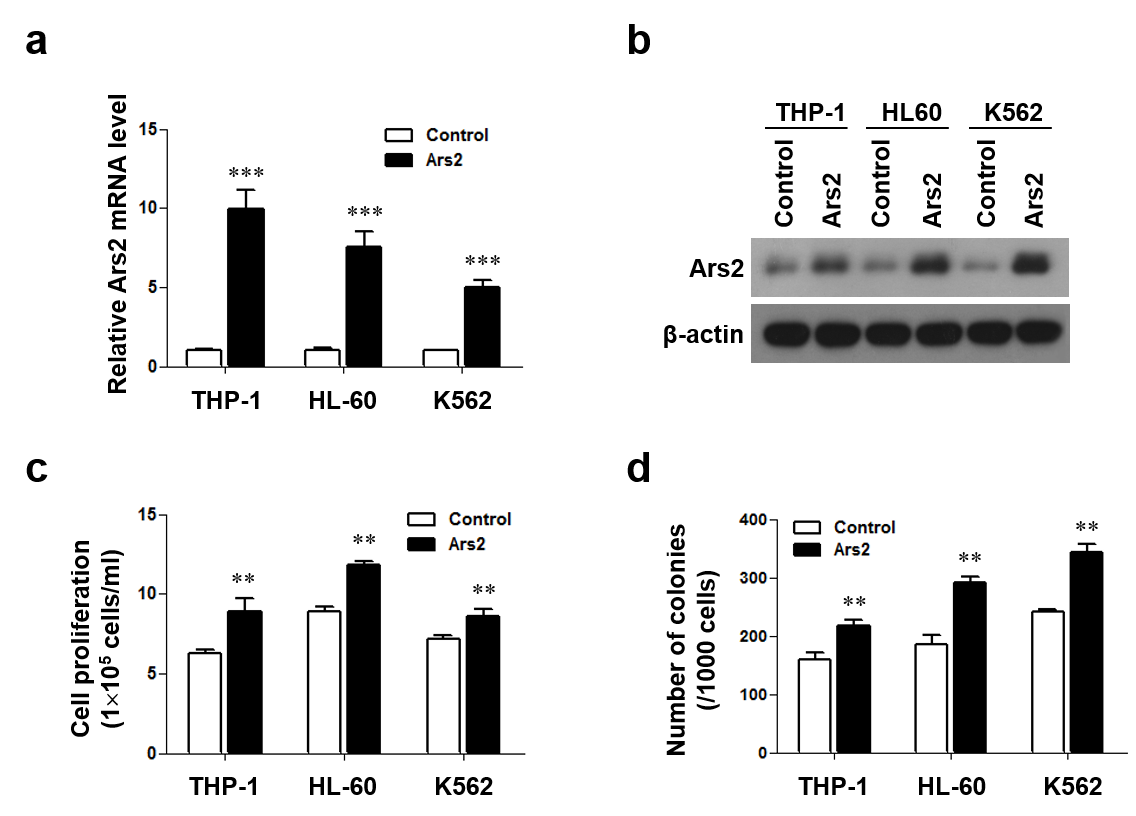
**

**Supplementary Figure 3.** Overexpression of Ars2 promotes cell proliferation and colony formation in AML cells. AML cell lines (THP-1, HL60, and K562) were transfected with either vector control or Ars2. (a and b) The expressions of Ars2 at mRNA and protein levels in THP-1, HL60, and K562 cells were detected by qRT-PCR and western blot analyses (****P* < 0.001). (c) Cell proliferation was detected using cell counting with Beckman Coulter Z2 Particle Counter. Data represent the means ± SD from three independent experiments (***P* < 0.01). (d) Colony formation was examined by staining colonies with 200 μl MTT per well. Colony number was counted using counter. The values represent the mean ± SD from three independent experiments. Values for Ars2-overexpressed cells are significantly higher than that for control cells by the Student’s *t* test; ***P* < 0.01.


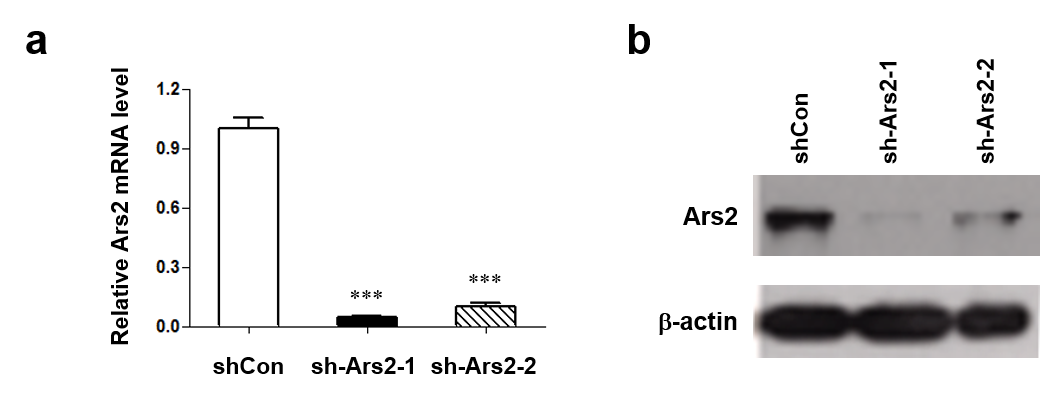


**Supplementary Figure 4.** Decreased expression of Ars2 in shArs2 cells. U937 cells were transfected with either vector control siRNA (shCon) or Ars2 siRNA (shArs2-1#, and shArs2-2#).

(a) The expression of Ars2 at mRNA levels in was detected by qRT-PCR analysis (****P* < 0.001 compared to shCon cells). (b) The expression of Ars2 at protein levels was detected by qRT-PCR and western blot analysis.

**
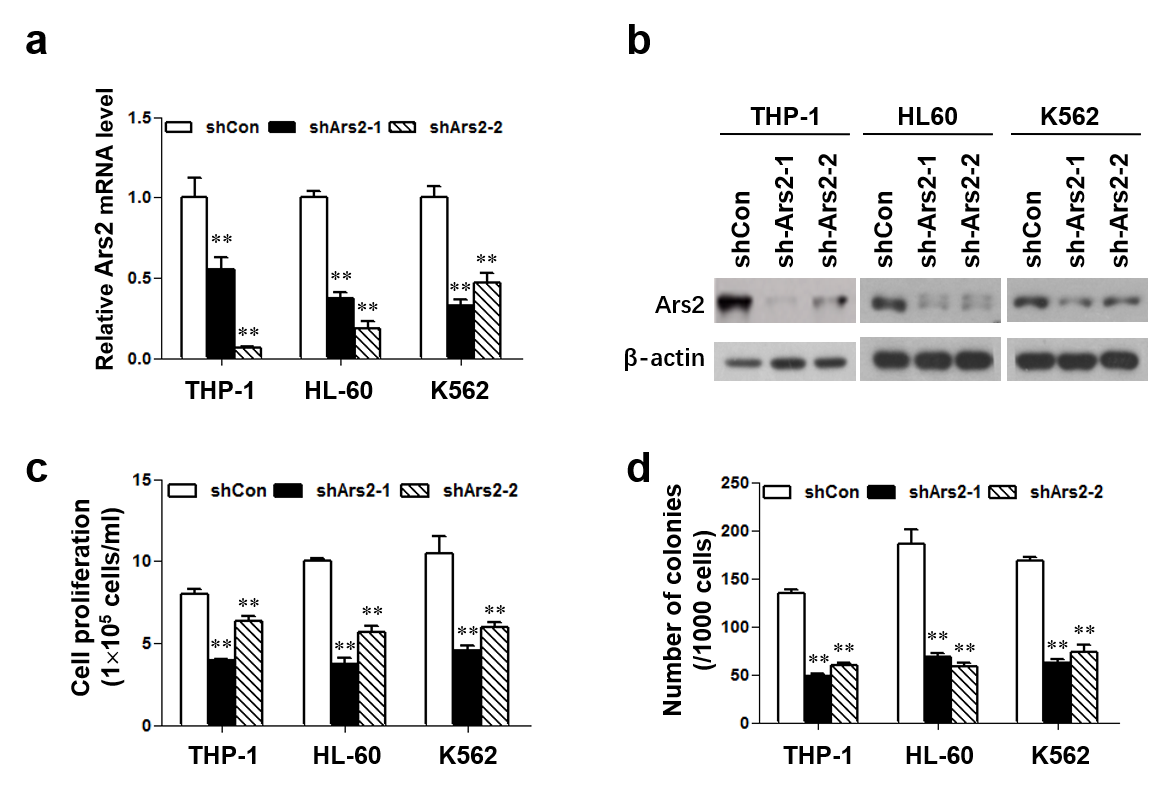
**

**Supplementary Figure 5.** Depletion of Ars2 suppresses cell proliferation and colony formation in AML cells. AML cell lines (THP-1, HL60, and K562) were transfected with vector control siRNA (shCon) and Ars2 siRNA (shArs2-1#, and shArs2-2#). (a and b) The expressions of Ars2 at mRNA and protein levels in THP-1, HL60, and K562 cells were detected by qRT-PCR and western blot analyses (***P* < 0.001). (c) Cell proliferation was detected using cell counting with Beckman Coulter Z2 Particle Counter. Data represent the means ± SD from three independent experiments (***P* < 0.01). (d) Colony formation was examined by staining colonies with 200 μl MTT per well. Colony number was counted using counter. The values represent the mean ± SD from three independent experiments. Values for shArs2 cells are significantly lower than that for shCon cells by the Student’s *t* test; ***P* < 0.01.

**
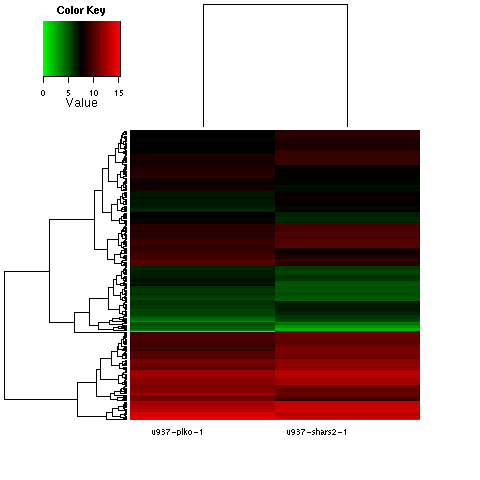
**

**Supplementary Figure 6.** miRNA microarray. U937 cells were transfected with either vector control shRNA (shCon) or shArs2, high-throughput screening of all genomics miRNAs was determined by RioArraymiDETECT MicroRNA Assay.


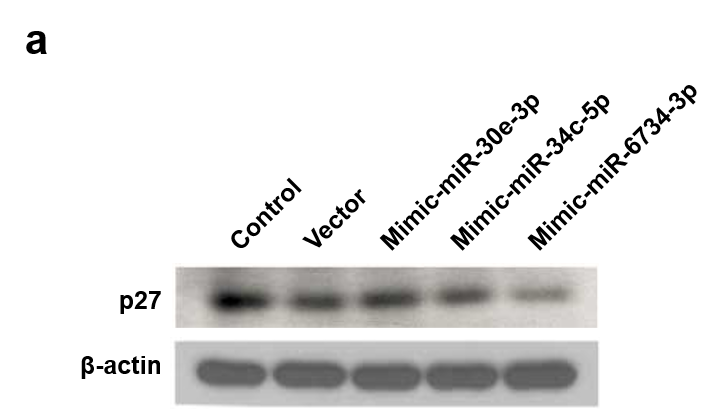


**Supplementary Figure 7.** miR-6734-3p mimic inhibits the expression of p27. U937 cells were transfected with vector or mimics for miR-6734-3p, miR-30e-3p and miR-34c-5p, after which expression of p27 at mRNA levels was determined by western blot analysis.


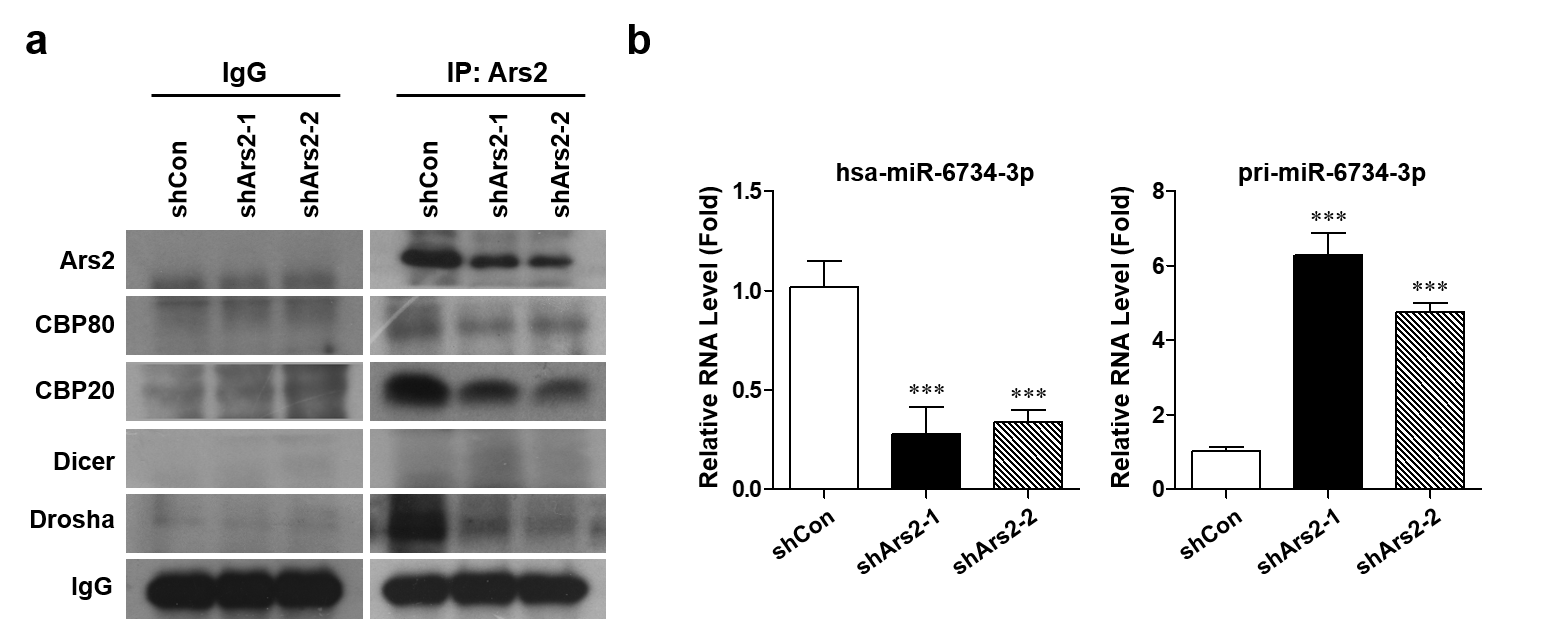


**Supplementary Figure 8.** Ars2 interaction with CBC is required for biogenesis of miR-6734-3p. (a) U937 cells were transfected with either shCon or shArs2 and immunoprecipitations were performed with anti-Ars2 beads. Following elution with Ars2 were fractionated by SDS-PAGE and detected by western blot using the antibodies indicated. (b) The expressions of miR-6734-3p and pri-miR-6734-3p in shCon and shArs2 cells were detected by qRT-PCR analysis.


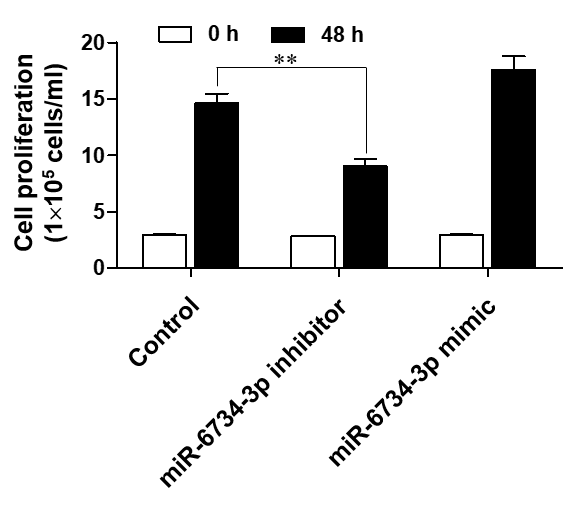


**Supplementary Figure 9.** The effects of miR-6734-3p inhibitor or mimic on cell proliferation in U937 cells. U937 cells were transfected with vector, miR-6734-3p inhibitor or miR-6734-3p mimic, after which cell proliferation was detected using cell counting with Beckman Coulter Z2 Particle Counter. Data represent the means ± SD from three independent experiments (***P* < 0.01).


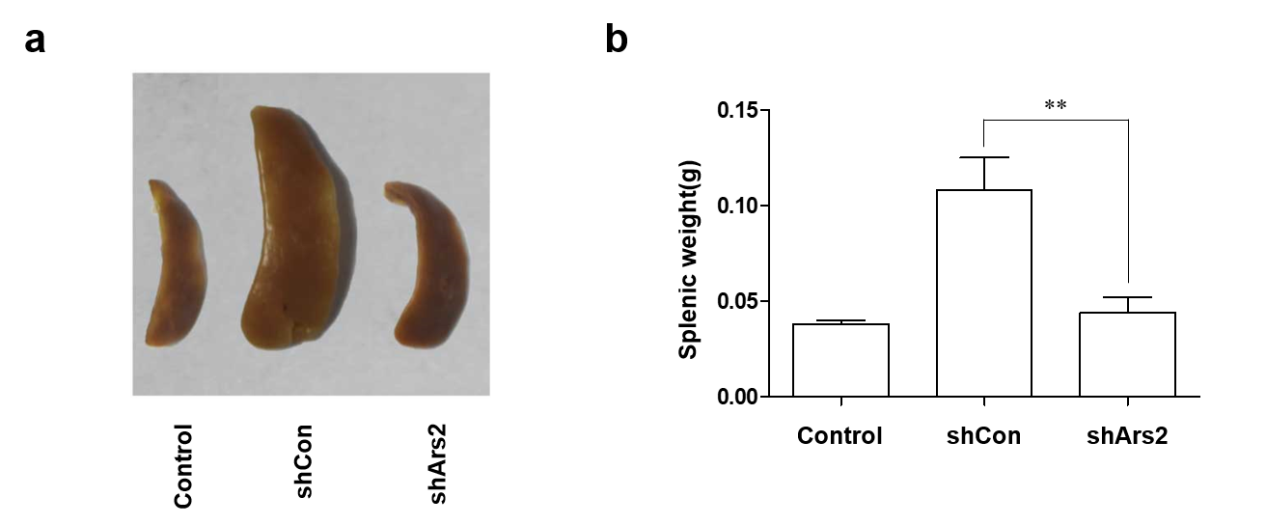


**Supplementary Figure 10.** Images and weights of spleens in scramble, shCon and shArs2 mice. (a) Images of spleens in scramble, shCon and shArs2 mice. (b) Spleem weights of individual mice. Data were represented as mean ± SD for 10 mice each group. *P* values were determined using two-tailed Student’s t-test (*P* < 0.01).

**
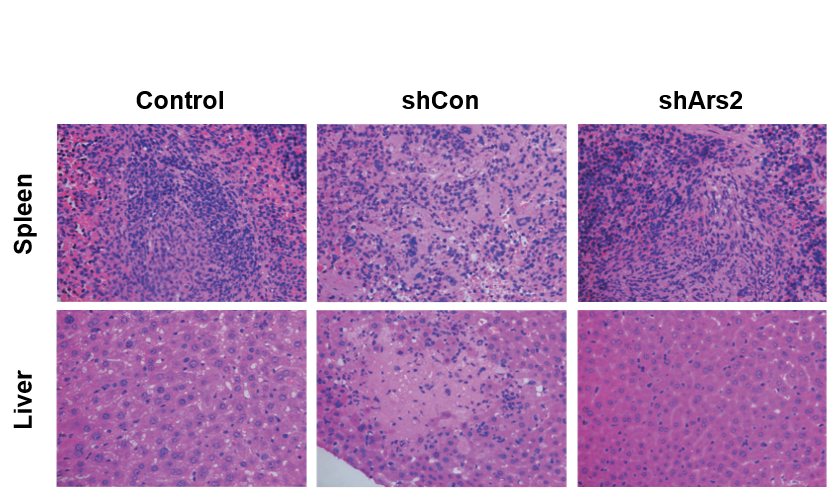
**

**Supplementary Figure 11.** Representative pictures of hematoxylin and eosin staining of livers and spleens from scramble, shCon and shArs2 mice.
